# Safety and efficacy of intradiscal nucleus pulposus allograft versus sham for lumbar discogenic pain associated with degenerative disc disease: study protocol for a randomized clinical trial

**DOI:** 10.1186/s13063-026-09826-w

**Published:** 2026-06-08

**Authors:** Timothy R. Deer, Pierce D. Nunley, Ramana K. Naidu, Douglas P. Beall, Timothy T. Davis, Aaron K. Calodney, Amol Soin, Morgan P. Lorio, Jon E. Block

**Affiliations:** 1 The Spine and Nerve Center of The Virginias, Charleston, WV USA; 2 Spine Institute of Louisiana, Shreveport, LA USA; 3 MarinHealth Spine Institute, Larkspur, CA USA; 4 Comprehensive Specialty Care, Edmond, OK USA; 5 Source Healthcare, Santa Monica, CA USA; 6 Precision Spine Care, Tyler, TX USA; 7 Ohio Pain Clinic, Centerville, OH USA; 8 Orlando College of Osteopathic Medicine, Winter Garden, FL USA; 9 Independent Consultant, San Francisco, CA USA

**Keywords:** Discogenic, Back pain, Lumbar, Intradiscal, Nucleus pulposus, Allograft

## Abstract

**Background:**

Chronic low-back pain is a leading cause of global disability, with degenerative disc disease (DDD) recognized as a common structural contributor. While conservative therapies may provide temporary symptom relief, they do not address the underlying degeneration of the intervertebral disc. Surgical interventions, such as spinal fusion or total disc replacement, are invasive procedures that permanently alter the anatomical structure of the vertebral motion segment. Minimally invasive intradiscal therapies have the potential to bridge the treatment gap between non-surgical management and surgery. Intradiscal delivery of nucleus pulposus (NP) allograft is intended to structurally supplement the degenerating disc and restore native disc function. Preliminary studies have suggested that a single intradiscal administration of NP allograft (VIA Disc NP) may improve pain and function in patients with lumbar discogenic pain.

**Methods:**

This is a randomized, double-blind, sham-controlled, multi-center clinical trial designed to evaluate the safety and efficacy of VIA Disc NP. Eligible participants are 22 to 85 years of age with MRI-confirmed lumbar DDD (modified Pfirrmann grade 3–7), axial low-back pain of at least 6 months’ duration, and functional impairment unresponsive to conservative treatment. Participants are randomized in a 2:1 ratio to receive either a single injection of VIA Disc NP or a sham procedure. The primary efficacy endpoint of this superiority trial is the proportion of participants achieving a ≥ 30% reduction in back pain severity at 12 months. Secondary endpoints include functional improvement (ODI), quality-of-life measures (EQ-5D-5L, PGIC), and opioid reduction. Sham participants who remain symptomatic at 12 months may cross over to receive active treatment.

**Discussion:**

This trial will provide level-1 evidence of the safety and efficacy of supplemental NP allograft therapy in patients with moderate to severe lumbar discogenic pain. If successful, this approach may offer a minimally invasive, durable, and structure-preserving treatment alternative to spinal fusion or disc arthroplasty in a population with limited therapeutic options.

**Trial registration:**

This trial is prospectively registered at ClinicalTrials.gov (Identifier: NCT06778447). Registered on January 16, 2025

**Supplementary Information:**

The online version contains supplementary material available at 10.1186/s13063-026-09826-w.

## Administrative information

Note: the numbers in curly brackets in this protocol refer to SPIRIT checklist item numbers. The order of the items has been modified to group similar items (see http://www.equator-network.org/reporting-guidelines/spirit-2013-statement-defining-standard-protocol-items-for-clinical-trials/).

**Table:** 

Title {1}	Safety and efficacy of intradiscal nucleus pulposus allograft versus sham for lumbar discogenic pain associated with degenerative disc disease: study protocol for a randomized clinical trial
Trial registration {2a and 2b}	This trial is prospectively registered at ClinicalTrials.gov (Identifier: NCT06778447).
Protocol version {3}	This manuscript is based on the final approved protocol: version 3.0, dated 13 May 2025.
Funding {4}	The trial is funded by Vivex Biologics, Inc. (Miami, FL, USA), the manufacturer of VIA Disc NP. The sponsor contributed to the design of the study, selection of clinical sites, and overall study governance.
Author details {5a}	Timothy Deer: The Spine and Nerve Center of The Virginias, Charleston, WV, USA Pierce Nunley: Spine Institute of Louisiana, Shreveport, LA, USA Ramana Naidu: MarinHealth Spine Institute, Larkspur, CA, USA Douglas Beall: Comprehensive Specialty Care, Edmond, OK, USA Timothy Davis: Source Healthcare, Santa Monica, CA, USA Aaron Calodney: Precision Spine Care, Tyler, TX, USA Amol Soin: Ohio Pain Clinic, Centerville, OH, USA Morgan Lorio: Orlando College of Osteopathic Medicine, Winter Garden, FL, USA Jon Block: Independent Consultant, San Francisco, CA, USA
Name and contact information for the trial sponsor {5b}	Leslie Zaccari, Vivex Biologics, Inc. (Miami, FL, USA)
Role of sponsor {5c}	The sponsor contributed to the design of the study; selection of clinical sites; collection, management, analysis, and interpretation of data; writing of the report; and the decision to submit the report for publication and overall study governance.

## Introduction

### Background and rationale {6a}

Lumbar discogenic pain associated with degenerative disc disease (DDD) contributes to substantial functional impairment and disability and has been identified as an important clinical target for therapeutic intervention [1, 2]. Degenerative changes within the nucleus pulposus (NP) contribute to reduced disc height, altered biomechanics, and pain symptomatology [3, 4]. Traditional nonoperative treatments—including physical therapy, oral medications, and corticosteroid injections—may offer temporary relief but do not address the underlying structural degeneration of the intervertebral disc. Surgical options, such as spinal fusion or total disc replacement, may be inappropriate for patients with moderately severe DDD where preservation of the vertebral motion segment is paramount. Consequently, there exists a significant treatment gap between conservative care measures and invasive surgical procedures in patients with chronic lumbar discogenic pain.

VIA Disc NP is a minimally manipulated, micronized NP allograft processed from donated cadaveric disc tissue and reconstituted in sterile saline at the time of the procedure. Delivered intradiscally under fluoroscopic guidance, it is intended to supplement the degenerated native nucleus with allogeneic matrix material while preserving spinal anatomy. Accumulating clinical evidence shows that this product may result in clinically meaningful improvements in pain, function, and quality of life through 2 years of follow-up following a single treatment [5, 6]. This investigation is designed to evaluate the safety and efficacy of VIA Disc NP in a multi-center, double-blind, sham-controlled randomized clinical trial.

### Objectives {7}

The primary objective is to evaluate the safety and efficacy of a single intradiscal injection of VIA Disc NP in participants with lumbar discogenic pain and magnetic resonance imaging (MRI)-confirmed DDD.

The study aims to determine whether VIA Disc NP supplementation improves pain and functional outcomes compared to sham control, as measured by the proportion of participants achieving a clinically meaningful reduction in back pain severity at 12 months.

Secondary objectives include assessing changes in disability, opioid use, quality of life scores, as well as tracking treatment-emergent adverse events (AE) over 24 months of follow-up.

### Trial design {8}

This is a randomized, double-blind, sham-controlled, multi-center clinical trial with an adaptive design and a planned enrollment of up to 446 participants, including up to 21 participants in an initial non-randomized roll-in phase. Eligible participants are randomized in a 2:1 ratio via permuted block design to receive either VIA Disc NP or sham procedure. Randomization is stratified by site and previous intradiscal biological treatment (Y/N). All participants, clinical personnel, and outcome assessors are blinded to group assignment.

The primary efficacy analysis of this superiority trial will be conducted at 12 months, with long-term efficacy, safety, and exploratory imaging data collected through 24 months. Pre-specified interim analyses will be used to guide the adaptive design while controlling type 1 error inflation and power.

Sham participants who meet crossover criteria at 12 months (visual analog scale (VAS) ≥ 40 mm and ODI ≥ 40) are eligible to receive VIA Disc NP and initiate a secondary 12-month follow-up. All procedures are standardized across sites and performed under moderate conscious sedation using a fluoroscopy-guided technique.

## Methods: participants, interventions, and outcomes

### Study setting {9}

The trial is conducted at up to 21 investigational sites across the USA, including academic and private clinical research centers with dedicated interventional spine capabilities. All sites must demonstrate operational readiness and procedural consistency during a roll-in phase prior to randomization. Investigators are board-certified or fellowship-trained in pain management, spine intervention, or a related specialty, and must be proficient in the administration of VIA Disc NP per the manufacturer’s instructions for use (IFU). Each site maintains the capacity for longitudinal follow-up and secure data management in accordance with good clinical practice (GCP) and Institutional Review Board (IRB) standards.

### Eligibility criteria {10}

Eligibility for inclusion is evaluated on a site-specific basis based on each participant’s medical, clinical, and radiographic presentation as well as for study consistency purposes.

To be eligible to participate in this study, participants must meet all of the following criteria:

1. Age 22 to 85 years old
2. Diagnosis of moderate to severe DDD on MRI, modified Pfirrmann grade 3–7
3. Chronic axial midline low back pain in the absence of lower extremity motor/sensory/reflex changes with or without referred non-radicular leg pain for at least 6 months prior to screening; unresponsive to at least 3 months of conservative care
4. Low back pain severity score of ≥ 40 to ≤ 90 mm on the VAS
5. Oswestry disability score (ODI) score of ≥ 40 to ≤ 80
6. Positive sustained hip flexion test
7. Demonstrated intolerance to sitting
8. Able to give voluntary, written informed consent to participate and have signed an informed consent form (ICF) specific to this study
9. Willing and able to comply with all study procedures and availability for the duration of the study with a life expectancy of > 2 years

Clinical notation: During initial physical examination screening, potential study participants will undergo a sustained hip flexion test (inclusion #6). This maneuver is undertaken with the patient in the supine, straight-leg position. Both legs are elevated simultaneously to approximately 45 degrees and then both are allowed to come down slowly. A positive response is determined by patient report of familiar pain and may be corroborated by examiner observation of guarding, facial grimace, or other pain behavior [7].

The sustained hip flexion test is a pragmatic, low-burden clinical maneuver used by investigators in this trial to help distinguish axial discogenic pain from other etiologies. Although not formally validated in large-scale studies, its inclusion reflects real-world practice among experienced interventionalists and may help enrich for patients with centrally mediated axial symptoms rather than peripheral radiculopathy. The protocol operationalizes the test with specific positional and temporal parameters to improve consistency across sites.

Participants who meet any of the following criteria will be excluded from participating in this study:

1. Contraindications to the proposed sedation/anesthetic protocol
2. Involvement of more than two lumbar discs as evidenced by 3 or more discs with a modified Pfirrmann grade of 3 or greater
3. Disc height of less than 4 mm for any disc between L1 and S1
4. Symptomatic vertebral compression fracture
5. Previous surgical treatment of the lumbar spine
6. History of sacroiliac (SI) joint fusion within the past 6 months
7. Received lumbar epidural or intradiscal steroid injection, lumbar facet-joint steroid injection, lumbar radiofrequency ablation, provocative or anesthetic discography, SI joint pain injection, injection of methylene blue, dextrose, glucosamine, and chondroitin sulfate, or biacuplasty within 3 months of the day 0 procedure. Received intraosseous radiofrequency nerve ablation procedure at the same or adjacent level (e.g., basivertebral nerve ablation or sinuvertebral nerve ablation)
8. Been a recipient of prior intradiscal stem cell/progenitor cell therapy or other biological intervention (e.g., mesenchymal stromal cells [MSC], platelet-rich plasma [PRP]) at the target intervertebral disc within 12 months of the day 0 procedure
9. Evidence of dynamic instability on lumbar flexion–extension radiographs (> 3 mm)
10. Grade 2 or higher spondylolisthesis at the target level, lumbar spondylitis or other undifferentiated spondyloarthropathy, or type III Modic changes adjacent to the target disc
11. Radiographic evidence of a full-thickness annular tear at the target disc or other abnormal disc morphology
12. Clinical suspicion of facet pain as primary pain generator
13. A systemic condition or disease not stabilized or judged by the investigator to be incompatible with participation in the study (e.g., current systemic infection, uncontrolled autoimmune disease, uncontrolled immunodeficiency disease, recent history of myocardial infarction, uncontrolled diabetes (> 7.0% HbA1C))
14. Received VIA Disc NP previously
15. Deemed unsuitable for clinical study participation by the investigator
16. Evidence of substance abuse (including marijuana); note: participants using prescribed extended-release narcotics (e.g., fentanyl patch, MS Contin, oxycontin) within the 3 months prior to screening and participants on long-acting opioids may be given an option to wean off opiates before enrollment; participants on short-acting opiates (e.g., hydrocodone, oxycodone, tramadol) may be included and utilization monitored after the treatment
  - Opioid use of more than 90 MME/day
17. Currently receiving treatment with radiation, chemotherapy, immunosuppression, or chronic steroid therapy (prednisone, or its equivalent, use of up to 5 mg/qd, or inhalation steroids for asthma is allowed)
18. Metal or ceramic implants in the lumbar spine region
19. Contraindications to MRI, including non-MRI compatible devices and active implantable devices such as spinal cord stimulators and intrathecal pumps
20. Involved in ongoing or closed (within 6 months of screening visit) litigation related to their back-pain condition
21. Any mental instability, unstable bipolar disorders, unmanaged post-traumatic stress disorder (PTSD) or uncontrolled anxiety/depression and/or require new or changed anti-depressants or anti-psychotic medications within 3 months of enrollment
22. Diagnosis of any traumatic neurological disorders that may impact the study as per the judgment of the investigator
23. Women who are pregnant or breastfeeding at the time of enrollment and/or plan to become pregnant during the study; pregnancy is confirmed by:
  - A positive pregnancy test during the screening visit
  - Self-reported pregnancy
24. Women of childbearing potential (WOCBP) who are not using a reliable form of contraception (as determined by the investigator)
25. Received any experimental drug or device to treat the same condition used within 6 months prior to the screening visit or during the course of the clinical trial
26. Other persistent pain/nerve issues including radiculopathy, leg pain, and cauda equina syndrome

### Who will take informed consent? {26a}

Patients with MRI-confirmed DDD will be screened for eligibility to participate in this study based on the above-mentioned criteria. After the patient has been assessed as eligible by the treating physician, he/she will receive initial study information. The research team at each clinical site conducts a detailed in-person consultation for eligible patients about all study stages, including information about the objectives, outcome measures, intervention characteristics, risks, benefits, and the confidentiality of data management. Data collection only begins after signing the ICF.

### Additional consent provisions for collection and use of participant data and biological specimens {26b}

This provision is not applicable since this trial does not involve collecting biological specimens for storage. The study’s primary and secondary outcomes use patient-reported outcome measures (PROMs), physical functional tests, and MRI measurements.

## Interventions

### Explanation for the choice of comparators {6b}

Participants randomized to the control arm will receive a sham procedure. The procedure will be identical to the investigational procedure with the following exception: a 20G spinal cannula will be carefully inserted through the skin and muscle of the back but will not penetrate the annulus fibrosus of the intervertebral disc(s). Sham procedures mimic setup, the procedure itself, and sedation but do not involve an intradiscal injection, and no saline or VIA Disc NP will be injected.

### Intervention description {11a}

Participants randomized to the investigational arm will receive VIA Disc NP. A single dose, intradiscal delivery of 100 mg of VIA Disc NP mixed with sterile saline will be administered according to product IFU. All investigators undergo IFU training and demonstrate procedural competency.

### Criteria for discontinuing or modifying allocated interventions {11b}

All treated participants will be followed for the pre-specified duration of the trial and all safety assessments will be completed. No enrolled participants will be exited from the study unless they withdraw consent or based on investigator decision for participant safety. Any participant withdrawn from the study for any reason will have their reason(s) for study discontinuation recorded. Participants may withdraw from the study after signing informed consent for any reason without penalty.

### Strategies to improve adherence to interventions {11c}

The treatment intervention under investigation is delivered in a single minimally invasive procedure at day 0 which does not require patient adherence to an intervention throughout the trial.

### Relevant concomitant care permitted or prohibited during the trial {11d}

A concomitant medication log will be used to track all medications (prescription and over-the-counter [OTC]) for each participant. The log will be kept current throughout the participant’s participation in the study and will document the following:

- Drug name
- Indication
- Start and stop dates
- Daily dosage and unit (i.e., mg, mL, tablet)
- Frequency
- Route
- Reason taken (i.e., adverse event)

A concomitant therapies log will be used to track all therapies (physical therapy, chiropractic care, acupuncture, massage therapy, etc.) for each participant. The log will be kept current throughout the participant’s participation in the study and will document the following:

- Therapy type
- Reason for therapy
- Start and stop dates
- Duration per session
- Frequency

Participants will be instructed to discontinue pain medications 48 h prior to the procedure. This excludes use of pain medicine other than acetaminophen and/or non-steroidal anti-inflammatory drugs (NSAIDs) for conditions unrelated to DDD.

All concomitant medications and therapies used within the 30 days prior to the screening visit, including medications used for chronic pain symptoms, will be documented on the respective electronic case report forms (eCRFs). Any initiation of new medications or changes to current medications during the course of the study should be recorded on the appropriate eCRF. No other experimental drug or device should be used within 6 months prior to the screening visit or during the course of the clinical trial.

Chronic pain medication including NSAID dosages will be required to remain stable or decrease throughout the trial. Any decreases must be undertaken in consultation with the investigator and documented appropriately in the medical records and appropriate study documents such as the concomitant medication log.

The following concomitant therapies are not permitted during the 24-month study period:

- Other intradiscal or facet injections
- Rhizotomies
- Surgery at the same and adjacent vertebral level(s)

If violations of concomitant therapies occur, these will be addressed in the statistical analysis through sensitivity analyses or exclusion from the per-protocol analysis data set.

### Provisions for post-trial care {30}

The participant will be instructed to limit strenuous activity for 72 h and only to perform the normal activities of daily life. After 72 h, normal activity level may be resumed within reason. Strenuous activities including bending, twisting, squatting, or flexing will be avoided until after the post-procedure appointment. Prescriptions may be given to the participant for medications as needed including steroids, muscle relaxers, or narcotic pain relievers. A subgroup of participants may experience moderate to severe pain after the procedure. Oral medications may be needed to treat this post-procedure pain and discomfort. An icepack may be given to the participant to place over the injection site in the event of post-procedure site discomfort.

A study completion form will be completed at the point the participant either completes the protocol-required follow-up or stops participation in the study prematurely to document information and reason for leaving the study including:

- Study completion
- Lost to follow-up
- Withdrawal of consent by participant
- Adverse event
- Protocol deviation
- Study exit due to other treatment
- Death
- Other

### Outcomes {12}

The primary efficacy endpoint is the proportion of participants who achieve a minimal clinically important difference (MCID), defined as at least a 30% reduction in back pain VAS score from baseline to 12 months, in the VIA Disc NP group compared to that in the sham control group [8, 9].

The primary safety endpoint will be the proportion of participants that experience one or more treatment-related (investigational product (IP) or procedure) adverse events (AE) in the VIA Disc NP group compared to the sham control group at 12 months.

Additional safety measures will include the following:

- Proportion of participants who experience an unexpected treatment-related serious adverse event
- Proportion of participants who experience any AE
- Proportion of participants who experience each unique AE

Secondary efficacy endpoints include:

- Mean change in VAS score for back pain severity assessed at each follow-up interval
- Mean change in ODI score for back function assessed at each follow-up interval
- Proportion of participants who achieve at least a 50% reduction in VAS score for back pain severity (substantial clinical benefit (SCB)) assessed at each follow-up interval [10]
- Proportion of participants who achieve a patient acceptable symptom state (PASS), defined as a VAS score for pain severity of 30 mm or less, assessed at each follow-up interval [11, 12]
- Proportion of participants who achieve a MCID, defined as a 30% or greater improvement in ODI score assessed at each follow-up interval [8, 9]
- Proportion of participants who achieve SCB, defined as a 50% or greater improvement in ODI score assessed at each follow-up interval [13]
- Proportion of participants who achieve a PASS, defined as a ODI score for back function of 20 or less at all follow-up intervals [14, 15]
- Proportion of participants in each ODI functional impairment category assessed at each follow-up interval [16]
- Mean change in EQ-5D-5L score assessed at each follow-up interval
- Mean PGIC score assessed at each follow-up interval

- For participants that have consented to opioid use assessment via prescription drug monitoring program (PDMP):
  - Reductions in opioid use, defined as a ≥ 30% decrease in total dispensed morphine milligram equivalents (MME) from baseline to all follow-up intervals

### Participant timeline {13}

**Fig. 1:**
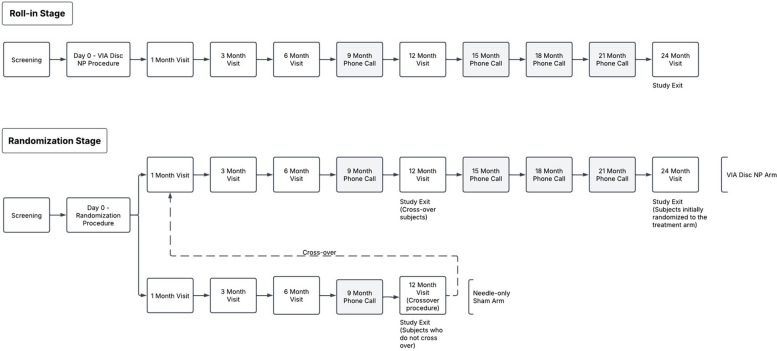
Participant timeline

Participants undergo screening and baseline assessments prior to the day 0 procedure. In-person visits are scheduled at months 1, 3, 6, 12, and 24 to collect efficacy and safety data. Phone check-ins at months 9, 15, 18, and 21 gather adverse event and medication updates. The 12-month visit includes crossover evaluation for sham participants (VAS ≥ 40 mm, ODI ≥ 40). All timepoints and procedures follow the protocol schedule of events (Table 1). The participant timeline is shown in Fig. 1.

**Table 1:** Schedule of enrollment, interventions, and assessments

Visit/assessment	Screening	VIA Disc NP procedure/randomization	1-Month visit	3-Month visit	6-Month visit	9-Month visit (phone call)	12-Month visit (sham arm crossover)	15-Month visit (phone call)	18-Month visit (phone call)	21-Month visit (phone call)	24-Month visit	Unscheduled visit
	(0–90 days prior*)	(Day 0)	(± 7 days)	(± 14 days)	(± 28 days)	(± 14 days)	(± 45 days)	(± 14 days)	(± 14 days)	(± 14 days)	(± 60 days)	
Informed consent	X^a^											
Eligibility criteria (inclusion/exclusion)	X											
Demographics and back treatment history	X											
Vital signs^b^	X	X										X
Medical history and labs (if applicable)	X											
Urine pregnancy test (for WOCBP)	X	X					X^h^					X^e^
Physical and neurological exams	X	X	X	X	X		X				X	X
MRI and evaluation	X^c^				X							
X-ray (less than or equal to 6 months from screening, AP, lateral, flexion–extension)	X^c^											
VIA Disc NP procedure		X^d^					X^h^					
Oswestry Disability Index (ODI)	X		X	X	X		X				X	
Visual analog scale (VAS)	X		X	X	X		X				X	X
EuroQol-5 Dimension (EQ-5D-5L)	X		X	X	X		X				X	
Patient global impression of change (PGIC)			X	X	X		X				X	
Concomitant medications	X	X	X	X	X	X	X	X	X	X	X	X
Opioid use assessment via PDMP							X					
Concomitant therapies	X	X	X	X	X	X	X	X	X	X	X	X
Adverse events	X	X	X	X	X	X	X	X	X	X	X	X
Study exit							X^g^				X	

*All effort should be made to complete screening within 14 days. Up to 90 days is provided in the event of delays to obtain MRI/MRS

^a^Study procedures must not be performed until after informed consent is signed

^b^Vital signs include oral temperature, sitting blood pressure, pulse oximetry, heart rate, and respiratory rate. On day 0 (day 0 visit), vital signs will be captured both pre- and post-procedure

^c^Participants will undergo MRI and X-rays within 6 months of screening unless site is obtaining advanced imaging, which requires new images. If greater than 6 months or not available, MRI and/or X-rays must be repeated. An MRI of the lower back per the imaging protocol must be obtained within 6 months (+ 28 days) of VIA Disc NP procedure

^d^Participants should be observed for adverse reactions for 30 min post-procedure prior to discharge

^e^Urine pregnancy test should be performed if the participant is a woman of childbearing potential and suspects pregnancy

^g^Participants who do not cross over at 12 months will exit the study

^h^Sham participants with VAS ≥ 40 mm and ODI ≥ 40 may cross over and receive the VIA Disc NP treatment at the 12-month visit

### Sample size {14}

The sample size for this study is based on a sham success rate assumption of 40% and varying superiority assumptions (delta) from 12.5 to 30 percentage points [17]. For initial study planning purposes, a frequentist *Z*-test for two proportions framework with pooled variance was used to calculate the required sample size to achieve 85% power using a one-sided alpha = 0.025 and 2:1 randomization.

Table 2 shows that when VIA Disc NP performance is 20 percentage points better than sham, *N* = 248 subjects are needed before loss to follow-up (LTFU). When VIA Disc NP performance is 15 percentage points better than sham, *N* = 446 subjects are needed (before LTFU). These sample sizes (*N* = 248 and *N* = 446) are the minimum and maximum number of subjects that will be enrolled (before LTFU).

**Table 2:** Range of sample size estimates based on MCID responder rate differences

Sham	Treatment	Delta	*N* (no LTFU)	*N* w/LTFU
40.0%	70.0%	+ 30.0%	107	119
40.0%	60.0%	+ 20.0%	248	276
40.0%	57.5%	+ 17.5%	326	362
40.0%	55.0%	+ 15.0%	446	496
40.0%	52.5%	+ 12.5%	641	712

### Recruitment {15}

The study will include adult participants, 22–85 years of age, with symptomatic moderate to severe DDD that is not adequately resolved by non-surgical standard care. Participants will be recruited through the clinical practices of participating investigators, IRB-approved local advertising, and referrals. Enrollment will commence in July 2025 and will continue until the required number of eligible participants is enrolled in the trial. Recruitment will take place at the aforementioned 20 institutions, where eligible participants will be identified and approached by trained research staff. Inclusion and exclusion criteria will be strictly followed. The recruitment period is expected to last 10 months.

## Assignment of interventions: allocation

### Sequence generation {16a}

Participants in the randomization stage will be randomized to the VIA Disc NP group or the sham control group in a 2:1 ratio via permuted block randomization with randomization stratified by clinical site and previous intradiscal biological treatment (Y/N). The randomization allocation schedule will be prepared in advance by an independent statistician not involved in the conduct of the trial. The randomization schedule will be generated using a validated randomization software (e.g., SAS, R, or another secure randomization tool).

Randomization will occur at the time of the day 0 visit after all eligibility criteria have been evaluated and the ICF is signed. Randomization may be performed using the appropriate module in the electronic data capture (EDC) system.

### Concealment mechanism {16b}

Each participant will be assigned a unique identification number upon enrollment. The randomization list will be securely stored and accessed only by authorized personnel responsible for assigning treatment codes. Treatment assignments (e.g., VIA Disc NP, sham injection) will be coded to ensure blinding and will be provided via a secure, web-based randomization system.

To protect against selection bias, the randomized treatment allocation is blinded. Thus, treatment assignment is concealed until the day of the procedure. This serves to eliminate the risk of pre-selecting certain patients for one group or the other by knowledge of upcoming treatment assignments.

### Implementation {16c}

The EDC system will only allow unblinded site personnel to complete the randomization form. They will be able to see the randomization treatment but blinded staff will only see the randomization number (no treatment assignment). Treatment allocation is provided on the day of the procedure by unblinded personnel to the unblinded physician performing the procedure.

## Assignment of interventions: blinding

### Who will be blinded {17a}

The following individuals will be blinded:

- Participants: when randomized into the trial, the patient will consent to be blinded to the type of treatment they will receive until the 12-month visit
- Outcome assessors
- Clinicians (sub-investigators) completing the physical or neurological examination
- Study coordinators administering PROMs and collecting other participant data

The treating investigator will be unblinded because they are performing the procedure; therefore, a different blinded sub-investigator will complete the follow-up assessments (physical exam, neurological exam) prior to unblinding at the 12-month visit. A blinded study coordinator may administer the questionnaires. The procedure case report forms (CRFs) for both treatment groups will be identical in the EDC system. IP accountability will be maintained separately. To assess the fidelity of the blinding, all participants will be queried immediately post-procedure and at the 12-month follow-up visit as to whether they believed they received the IP or sham.

### Procedure for unblinding if needed {17b}

When it is necessary to break the blind prematurely, the researcher will be required to notify the IRB. The sponsor will be notified as soon as possible, preferably by email or telephone and then in writing, regarding the necessity of code breaking.

Blinded site personnel (e.g., clinical study coordinator, sub-investigators) will record the participant’s PROM scores at the 12-month visit. All participants will then be unblinded to original treatment assignment. Unblinding for purposes of crossover will occur as follows:

- Participants randomized to the sham control group with VAS ≥ 40 mm and ODI ≥ 40 at 12 months will be approved for crossover to VIA Disc NP treatment and followed for an additional 12 months.
- Participants in the sham control group with VAS < 40 mm or ODI < 40 at 12 months will be informed that they do not meet the criteria for crossover and will exit the trial.

All participants will proceed unblinded after the 12-month visit to study completion at 24 months.

Emergency unblinding may be necessary if a participant experiences a serious adverse event (SAE) that requires knowledge of the treatment assignment for appropriate medical management. The investigator will contact the designated unblinding contact (e.g., study monitor or medical monitor) to request unblinding. The request for unblinding will be documented, including the reason for unblinding and the date and time of the request. The unblinding contact will access the randomization list and provide the treatment assignment to the investigator. The unblinded information must be treated confidentially and shared only with those necessary for the participant’s safety.

If any blinded party becomes unblinded to treatment allocation for a particular participant, this should be recorded using the unblinding CRF. If a participant is unblinded, they will stay in the trial and be followed up as usual.

Planned unblinding will occur after all primary endpoint data have been collected at the 12-month visit. The process for planned unblinding will be documented in the statistical analysis plan (SAP). The unblinding process will be overseen by the study statistician and data management team. Once unblinding occurs, the treatment assignments will be revealed to the investigators, study team, and participants, as appropriate.

## Data collection and management

### Plans for assessment and collection of outcomes {18a}

The study will be monitored to ensure that it is conducted in conformance with the monitoring plan to assess continued compliance with the protocol, recognized Good Clinical Practices, and US federal regulations outlined in 21 CFR Part 312. In addition, the monitor will verify that study records are adequately maintained; that data are reported in a satisfactory manner with respect to timeliness, adequacy, and accuracy; and that the investigator continues to have sufficient staff and facilities to conduct the study safely and effectively.

The study may also be participant to a quality-assurance audit by the sponsor or its designees as well as inspection by appropriate regulatory authorities. In addition to ensuring adequate communication between the investigators and the sponsor, the monitor’s duties include on-site and remote visits (as necessary) and review of study documents and reported data. The sponsor representatives will be provided with appropriate product and protocol training prior to the study and will follow a monitoring plan for all study-related monitoring activities.

On-site and remote monitoring visits will include a pre-study site initiation visit, periodic interim monitoring visits, and a close-out visit at the end of the site’s participation in the study.

Monitoring visits will be documented on monitoring visit reports and will aim to verify that:

- Compliance with the clinical protocol and applicable regulations is being maintained.
- Source data are verified and signed off upon as accurate.
- Participant files are accurate and complete.
- Participant withdrawal has been documented (if applicable).
- Participant non-compliance has been documented (if applicable).
- The investigator and site staff are informed and knowledgeable of all relevant document updates concerning the clinical investigation.
- Only authorized individuals are performing study-related functions.
- The IP is being used according to the protocol and instructions for use.
- Staffing and facilities are adequate.
- Signed and dated ICFs have been obtained from each participant.
- CRFs and queries are complete.
- All AEs are reported to the sponsor.
- All SAEs and unanticipated adverse events (UAEs) are reported to the sponsor and the IRB.
- All other required IRB reports, notifications, applications, submissions, and correspondence are maintained in the investigator’s files and are accurate.
- Corrective and preventive actions have been implemented (if applicable).

To ensure that the study is conducted in accordance with the terms of the clinical protocol, study monitors must visit each study site at routine intervals throughout the duration of the study. The exact frequency of visits shall be determined on an individual study site basis as detailed in the clinical monitoring plan and shall depend upon the following factors:

- Rate of participant enrollment
- Experience of the investigator in conducting clinical studies
- Record of previous site compliance

eCRFs will be completed in a 21 CFR Part 11-compliant EDC system for each participant consented into the clinical study. The study coordinator will enter the source data onto the CRF, and it will then be entered into the EDC and study monitors will verify that the source documents match the data entered on the CRF and in the EDC. Once verified, the study monitor will lock the field or page via a monitoring approval step so the data cannot be changed unless requested.

CRFs will be completed on paper forms for each participant consented for the clinical study. The study coordinator will enter the source data onto the CRFs, and data from the CRF pages will be entered into the EDC system. Study monitors will verify that the source documents match the CRFs at selected time intervals.

All procedures for the handling and analysis of data will be conducted using good clinical data management practices within systems meeting US Food and Drug Administration (FDA) guidelines for the handling, storage, and analysis of data for clinical studies.

The investigator will sign off on the eligibility criteria, all adverse event, protocol deviation, and study completion CRFs and will sign each participant’s casebook once the participant completes the study to attest that all data entered on the CRFs are complete and accurate.

Any required data clarifications will be handled within the EDC system’s query management system. The requests will include but not be limited to queries on missing values and values out of acceptable ranges. Study coordinators will have the opportunity to correct data and/or respond to the query for review by monitors and data managers.

PROMs will be collected via paper forms and the data will be transmitted into the EDC. Any data that are originated on paper are to be considered source documentation for the purposes of this study. The data quality control measures for PROM data entry will include double data entry and automated checks in the EDC system, to minimize transcription errors.

Each participant will be identified in the database by a unique participant identifier assigned in EDC. To ensure the quality of clinical data across all participants and sites, a clinical data management review will be performed on participant data according to specifications outlined in the data management plan. Data will be vetted both electronically and manually. Queries generated and raised by reviewers will be generated within the EDC application. During this review, participant data will be checked for consistency, omissions, and any apparent discrepancies.

All AEs will be coded using the medical dictionary for regulatory activities (MedDRA), and all concomitant medication will be coded using the World Health Organization Drug Reference (WHODRUG Global) List*.*

### Plans to promote participant retention and complete follow-up {18b}

The participants will receive extensive information about the study methods, setup, and requirements during the recruitment phase. The importance of completion of the follow-up assessments will be emphasized. Patients will be allowed to withdraw from study participation at any time during the study and are not obliged to give a reason for discontinuation. All participants will be reminded throughout the study to fill out the PROMs during study visits. Throughout the follow-up period, the researchers will check responses and if necessary, contact patients for completion of their follow-up.

### Data management {19}

Each participant will be identified in the database by a unique participant identifier assigned in EDC. To ensure the quality of clinical data across all participants and sites, a clinical data management review will be performed on participant data according to specifications outlined in the data management plan. Data will be vetted both electronically and manually. Queries generated and raised by reviewers will be generated within the EDC application. During this review, participant data will be checked for consistency, omissions, and any apparent discrepancies.

### Confidentiality {27}

The investigators and the sponsor will preserve the confidentiality of all participants taking part in the study, in accordance with GCP and local regulations. The investigator will ensure that the participant’s anonymity is maintained. CRFs or other documents submitted to the sponsor will identify each participant by a unique participant identifier as designated by the sponsor. Documents that are not for submission to the sponsor (e.g., signed ICF) will be kept in strict confidence by the investigator.

In compliance with federal regulations/ICH GCP guidelines, it is required that the investigator and institution permit authorized representatives of the company, of the regulatory agency(s), and the IRB direct access to review the participant’s original medical records for verification of study-related procedures and data. The investigator is obligated to inform the participant that his/her study-related records will be reviewed by the above-named representatives without violating the confidentiality of the participant.

### Plans for collection, laboratory evaluation, and storage of biological specimens for genetic or molecular analysis in this trial/future use {33}

No biological specimens will be collected in this trial.

## Statistical methods

### Statistical methods for primary and secondary outcomes {20a}

Descriptive analyses will be performed to characterize the treatment groups and to assess equality produced in reality from the randomization to understand if no clinically significant group differences at baseline exist. Baseline variables will be compared between groups using means, standard deviations, median, minimum, and maximum values for continuous variables and count and percentages for categorical variables.

Although the emphasis will be on clinical significance, baseline comparisons will include *t*-tests or Wilcoxon rank-sum tests as appropriate for interval variables and chi-square or Fisher’s exact tests as appropriate for nominal variables to aid in the screening for baseline differences.

Changes in clinical endpoints over time will be summarized within each treatment group using summary statistics, including mean and median change scores, standard deviations, and ranges. Pearson or Spearman rank correlation coefficients will be used to characterize associations among variables within the treatment groups. For time-to-event outcomes (i.e., event-free survival), descriptive analyses will include the construction of group-specific Kaplan–Meier survival curves as appropriate. Longitudinal clinical status will be evaluated for all PROMs.

The analysis populations for this study will include the intent-to-treat (ITT) analysis set, modified intent-to-treat (MITT) analysis set, safety analysis (SA) set, and per protocol (PP) analysis set.

The primary efficacy measurements will be characterized descriptively in terms of the proportion of participants meeting at least a 30% pain improvement from baseline in VAS back-pain severity score at 12 months, in the VIA Disc NP group compared to that in the sham control group.

The claim of superiority, for the primary endpoint of month 12 VAS 30% improvement, will be accepted if the posterior probability of superiority is greater than or equal to 0.975. That is, superiority will be claimed if:

$$\mathrm{PR}(\mathrm{pT}-\mathrm{pC}>0\ |\ \mathrm{Data}) \geq <span class='crossLinkCiteEqu'>0</span>.975$$

This posterior probability is calculated using beta non-informative Jeffreys priors for both groups. Participants who receive alternative treatments during the study, e.g., epidurals or increased narcotics, will be handled analytically in the primary endpoint calculation. In the same fashion, participants who do not complete the study will be categorized by the reason for missingness and handled according to this designation. These events (additional treatments or exiting the study for specific reasons) are called intercurrent events. At a high level, participants with temporally proximate, clinically meaningful alternative treatments will have their month 12 endpoint data imputed from participants without additional treatment. This is consistent with ICH E9R1 discussion and the methodology of defining estimates of interest and intercurrent events.

For the VAS, ODI, EuroQol (EQ-5D), and patient global impression of change (PGIC) endpoints, mean change in scores at each follow-up time-analysis will be performed with the hypothesis:

H0: dt − dc ≤ 0.

Ha: dt − dc > 0.

where “dpt” is the mean change in VAS and ODI scores among the treatment (dt) and control (dc) arms, respectively. Endpoints defined as proportions will be tested by comparing differences in proportions. The tests will be conducted with the hypotheses at each follow-up interval:

H0: pt − pc ≤ 0.

Ha: pt − pc > 0.

where “pt” and “pc” are the percentage of responders in the treatment (pt) and control arms (pc), respectively, at 12 months. These analyses will be conducted for all differences in proportion endpoints including:

- Proportion of participants who achieve at least a 50%-reduction in VAS score for back-pain severity SCB assessed at each follow-up interval
- Proportion of participants who achieve a PASS, defined as a VAS score for pain severity of 30 mm or less, assessed at each follow-up interval
- Proportion of participants who achieve a MCID, defined as a 30% or greater improvement in ODI score assessed at each follow-up interval
- Proportion of participants who achieve SCB, defined as a 50% or greater improvement in ODI score assessed at each follow-up interval
- Proportion of participants who achieve a PASS, defined as a ODI score for back function of 20 or less at all follow-up intervals
- Proportion of participants in each ODI functional impairment category assessed at each follow-up interval

Secondary endpoints will be tested using both Bayesian and frequentist statistics.

- For participants that have consented to opioid use assessment via PDMP:
  o Reductions in opioid use, defined as a ≥ 30% decrease in total dispensed MME from baseline to all follow-up intervals

A cumulative proportion of responders analysis (CPRA) will be used to robustly compare the proportion of responders achieving clinical success in ODI and VAS endpoints. Group-specific vectors of month 12 change from baseline scores will be generated. Those vectors represent group-specific cumulative proportions of responders and will be statistically compared using a 2-sample Kolmogorov–Smirnov test. The CPRA will be visualized as follows:

- X-axis details the point change from baseline in the PRO score at month 12.
- The Y-axis details the group-specific percentage of subjects achieving the corresponding x-axis’s value, 0 to 100%, where each group will start in the upper left-hand of the graphic depicting 100% of participants.

The groups include the VIA Disc NP treatment arm in blue and the control arm in red at every group-specific unique change from baseline, represented by a dot, along the group-specific distribution function.

### Interim analyses {21b}

Pre-specified interim analyses will be used to guide the adaptive design while controlling type 1 error inflation and power.

### Methods for additional analyses (e.g., subgroup analyses) {20b}

Exploratory pre-specified subgroup analysis will be performed. The primary endpoint will be repeated stratified by each subgroup. The primary endpoint and the prongs of the CCS will be reported. The stratifications will be done within treatment arm and an exact *p* value with associated 95% confidence interval will be reported. Additional analyses will be done as appropriate for additional clinical context.

1. Age (split at median)
2. Sex
3. Number of vertebral level(s) treated (1 vs. 2)
4. Baseline Pfirrmann grade
5. Baseline Modic changes

### Methods in analysis to handle protocol non-adherence and any statistical methods to handle missing data {20c}

Participants lost to follow-up may produce bias in this study. Reasons for discontinuation will be evaluated and characterized. Participants without month 12 data will be included through statistical imputation. The participants will be included in the analysis using Bayesian multiple imputation which may include covariates to aid in the imputation, including age, sex, body mass index, and race/ethnicity.

A sensitivity analysis will be conducted and is an important tool for additional characterization of missing data. A tipping point analysis will be provided to characterize the robustness of the primary study results. The tipping point analysis assigns every combination of success and failure to each participant missing the CCS. The tipping point makes no assumptions on the reason for missingness.

### Plans to give access to the full protocol, participant-level data, and statistical code {31c}

In the event of a study-specific publication, the subject-level datasets and derived variables used and/or analyzed during the trial will be made available, subject to any existing ethical or legal constraints, by the corresponding author upon reasonable request. The data sharing plan will adhere to the standard clinical trial reporting guidelines.

## Oversight and monitoring

### Composition of the coordinating center and trial steering committee {5d}

The clinical site of the principal investigator is the Spine and Nerve Centers of the Virginias (Charleston, WV, USA). This trial does not have a steering committee.

### Composition of the data monitoring committee, its role and reporting structure {21a}

This trial employs two independent medical monitors to adjudicate all adverse events and deviations from protocol.

### Adverse event reporting and harms {22}

An AE is any untoward medical occurrence in a participant or clinical investigation participant administered a pharmaceutical product and which does not necessarily have to have a causal relationship with this treatment. An AE can therefore be any unfavorable and unintended sign (including an abnormal laboratory finding, for example), symptom, or disease temporally associated with the use of a medicinal product, whether or not considered related to the medicinal product.

It is the responsibility of investigators, based on their knowledge and experience, to determine those circumstances or which should be considered adverse events.

A serious AE (SAE) is any untoward medical occurrence that:

- Results in death,
- Is life-threatening,
- Requires inpatient hospitalization or prolongation of existing hospitalization,
- Results in persistent or significant disability/incapacity,
- Is a congenital anomaly/birth defect, or
- Is an important medical event.

Note: The term “life-threatening” in the definition of “serious” refers to an event in which the participant was at risk of death at the time of the event; it does not refer to an event which hypothetically might have caused death if it were more severe.

Medical and scientific judgment will be exercised in deciding whether expedited reporting is appropriate in other situations, such as important medical events that may not be immediately life-threatening or result in death or hospitalization but may jeopardize the participant or may require intervention to prevent one of the other outcomes listed in the definition above. Examples include allergic bronchospasm, convulsions, and blood dyscrasias or development of drug dependency or drug abuse.

Note:

- A procedure is not an AE or SAE, but the reason for the procedure may be an AE or SAE.
- Pre-planned (prior to signing the ICF) procedures or treatment requiring hospitalizations for pre-existing conditions that do not worsen in severity are not SAEs.

Unexpected AE (UAE) or suspected adverse reaction refers to an event or reaction that is not listed in the investigator’s brochure or is not listed at the specificity or severity that has been observed; or, if an investigator’s brochure is not required or available, is not consistent with the risk information described in the general investigational plan or elsewhere.

Possible adverse events may include:

- Bladder or bowel dysfunction
- Cerebrospinal fluid fistula
- Disorder of potency or disorder of sexual sensibility
- Epidural bleedings
- Hematoma
- Immunologic response (the possibility that a patient may develop alloantibodies should be considered for any patient who might be a future recipient of allograft tissue or cells)
- Infections (from local infections such as superficial wound infections up to spondylodiscitis and/or meningitis)
- Neurological deterioration as serious as lesions of the cauda equina such as transient radiculopathy, cauda equina syndrome, or paraplegia secondary to ascending bleeding or infection
- Pain and/or inflammation
- Paresis
- Relapsing herniation
- Sensory disturbances
- Transmission of disease of unknown etiology and transmission of infectious agents including but not limited to HIV, hepatitis, syphilis, or microbial contaminants
- Vertebral end plate inflammation

The following definitions should be used to assess intensity of AEs:

- Mild: Awareness of sign or symptom, but easily tolerated, i.e., does not interfere with participant’s usual function.
- Moderate: Discomfort enough to cause interference with usual activity.
- Severe: Incapacitating with inability to work or do usual activity, i.e., interferes significantly with participant’s usual function.

The investigator will assess causal relationship between an AE and the study product on the basis of his/her clinical judgment and the following definitions. The causality assessment will be made based on the available information and can be updated as new information becomes available.

- Not related
- Possibly related
- Probably related
- Definitely related
- Unknown

AE disposition will be judged as follows:

- 1 = recovered/resolved
  - The participant fully recovered from the AE with no residual effect observed.
- 2 = recovered/resolved with sequelae
  - The residual effects of the AE are still present and observable.
  - Include sequelae/residual effects.
- 3 = not recovered/not resolved
  - The adverse event itself is still present and observable.
- 4 = recovering or resolving
- 5 = fatal
- 6 = unknown

Clinical actions required can include:

- 1 = none
  - No treatment was required.
- 2 = medication required
  - Prescription and/or over the counter medication was required to treat the AE.
- 3 = hospitalization or prolongation of hospitalization required
  - Hospitalization was required or prolonged due to the AE, whether or not medication was required.
- 4 = other

An SAE will be reported to the sponsor within 24 h of the study site becoming aware of the event and entered into the EDC system. The investigator will provide additional information on the SAE by updating the information on the CRFs and in the EDC as updates become available and transmitted to the sponsor. The sponsor may also ask for additional clinical reports including redacted source documents to be provided by the investigator to assist in the assessment of the event. Significant new information and updates will continue to be submitted promptly to the sponsor, entered on CRF pages, and in the EDC as they become available, and the investigator will follow the SAE until it is resolved, or no further improvement is expected.

The sponsor ensures that the investigator submits safety event notifications to the governing IRB within the timeframe specified by the IRB, when applicable. Acceptable means of confirming that the IRB requirements have been met include forwarding a copy of the written, signed report that was sent to the IRB to the sponsor. Copies of this report should be filed in the investigator’s site files and the sponsor’s clinical investigation files.

New SAEs will only be documented for each participant until the last study-related participant visit. In case of acute risk, the incident must be reported immediately.

### Frequency and plans for auditing trial conduct {23}

The investigator/investigational site will permit study-related monitoring, audits, IRB review, and regulatory inspections by providing direct access to source data/documents. Direct access includes permission to examine, analyze, verify, and reproduce any records and reports that are important to the evaluation of a clinical study. The investigator/designees will be responsible for obtaining and gathering information related to unexpected AEs and SAEs and reporting those as related to VIA Disc NP to their IRB, and to the product manufacturer.

### Plans for communicating important protocol amendments to relevant parties (e.g., trial participants, ethical committees) {25}

Any amendment to the study protocol that is deemed to be appropriate as the study progresses will be communicated to the investigator by the sponsor. All protocol amendments will undergo the same review and approval process as the original protocol. A protocol amendment may be implemented after it has been approved by the IRB. The sponsor will assure the timely submission of amendments to regulatory authorities.

### Dissemination plans {31a}

The clinical trial agreement between the sponsor and the study site and investigator shall govern the publication rights of the parties. The data provided by this clinical trial, and all related information and any materials containing such data and information, are the exclusive property of the sponsor and are confidential. The investigator or other study-related personnel may not disclose to anyone or use any data, information, or materials related to this clinical trial without the express prior written consent of the sponsor.

The sponsor may, in its sole discretion, publish the findings of the study in the peer-reviewed medical literature. Publication of the results will be based upon appropriate analyses and review of the complete data and will occur primarily under the direction of the sponsor, which will develop guidelines for authorship inclusion. Neither the investigator nor any other third party may publish the results of the study or any related information without the prior written consent of the sponsor in compliance with the executed clinical study agreement between such parties. The plan for publication of data is to be within 12 months of trial completion.

## Discussion

The successful execution and completion of this trial protocol will provide level 1 evidence of safety and efficacy of intradiscal NP allograft for lumbar discogenic pain associated with DDD. The use of a sham control group preserves the high methodological quality of this investigation. Previous clinical research suggests that a single intradiscal administration of NP allograft provides durable pain amelioration through 2 years of post-procedural follow-up [6]. If confirmed, this treatment approach using a commercially available, off-the-shelf product could have broad and substantial healthcare impact, given the high prevalence of chronic low back pain worldwide. Bridging the treatment gap between conservative care and invasive surgical procedures, intradiscal NP allograft may offer the advantage of preserving the natural kinematics of the intervertebral disc without structural changes to the anatomy of the vertebral motion segment.

Although this randomized, double-blind, sham-controlled, multi-center trial is robustly designed, there are limitations including challenges in maintaining blinding, analytical complexities from the crossover design, restricted generalizability due to strict eligibility criteria, and reliance on subjective PROMs. For example, while an interim analysis of 170 cases found that midline low-back pain exacerbated by sustained hip flexion is a strong predictor of lumbar discogenic pain, this physical examination eligibility component has not been validated in this setting [7]. Additional concerns involve untested assumptions in sample size calculations and limited long-term safety data. While the protocol incorporates mitigations like blinded assessors, imputation for missing data, and interim analyses, these limitations may affect the trial’s external applicability.

## Trial status

Enrollment commenced in July 2025 and will continue until the required number of eligible participants is enrolled in the trial which is expected to take 10 months. The participants will be followed for 24 months and an estimated timeline for the trial including subject recruitment, follow-up, and data analysis will be approximately 30 months with expected completion of the trial by December 2027.

## Supplementary Information

Supplementary Material 1.

## Data Availability

The datasets generated during the study will be available after publication of the trial results upon written request made to the corresponding author.

## Abbreviations

AE: Adverse events
CRF: Case report form
DDD: Degenerative disc disease
eCRF: Electronic case report form
EDC: Electronic data capture
EQ-5D: EuroQol
FDA: Food and Drug Administration
GCP: Good clinical practice
ICF: Informed consent form
IFU: Instructions for use
IP: Investigational product
IRB: Institutional review board
ITT: Intention to treat
LTFU: Loss to follow-up
MedDRA: Medical dictionary for regulatory activities
MCID: Minimal clinically important difference
MI: Multiple imputation
MME: Morphine milligram equivalents
MRI: Magnetic resonance imaging
MRS: Magnetic resonance spectroscopy
MSC: Mesenchymal stromal cell
NP: Nucleus pulposus
NSAID: Non-steroidal anti-inflammatory drug
ODI: Oswestry Disability Index
OTC: Over the counter
PASS: Patient acceptable symptom state
PDMP: Prescription drug monitoring program
PGIC: Patient global impression of change
PP: Per protocol
PRP: Platelet-rich plasma
PTSD: Post-traumatic stress disorder
SAE: Serious adverse event
SAP: Statistical analysis plan
SCB: Substantial clinical benefit
SI: Sacroiliac
UAE: Unanticipated adverse event
VAS: Visual analog scale
WOCBP: Woman of childbearing potential
