# Supplementary material for: Safety and efficacy of intradiscal nucleus pulposus allograft versus sham for lumbar discogenic pain associated with degenerative disc disease: study protocol for a randomized clinical trial
Source: Trials. 2026 Jun 8;27:526. doi: 10.1186/s13063-026-09826-w (PMC13471482; doi:10.1186/s13063-026-09826-w)
Supplement: Supplementary file 1 [file 13063_2026_9826_MOESM1_ESM.docx]

**RESEARCH SUBJECT CONSENT FORM**

**TITLE:**	Randomized, Multi-Center, Double-Blind Clinical Trial Evaluating the Safety and Efficacy of Nucleus Pulposus Allograft to Supplement Nucleus Pulposus Tissue in Participants with Lumbar Discogenic Pain Associated with Degenerative Disc Disease (RESTORE)

**PROTOCOL NO.:**	VIA-2024-002

WCG IRB Protocol #20244769

**SPONSOR:**	Vivex Biologics, Inc.

**INVESTIGATOR:**	Name

Address

City, State, Zip

Country

**STUDY-RELATED**

**PHONE NUMBER(S):**	Phone Number

Phone Number (24 hours)

[24-hour number is required]

Taking part in this research is voluntary. You may decide not to participate, or you may leave the study at any time. Your decision will not result in any penalty or loss of benefits to which you are otherwise entitled.

If you have any questions, concerns, or complaints or think this research has hurt you, talk to the research team at the phone number(s) listed in this document.

# RESEARCH CONSENT SUMMARY

You are being asked for your consent to take part in a research study. This document provides a concise summary of this research. It describes the key information that we believe most people need to decide whether to take part in this research. Later sections of this document will provide all relevant details.

# How long will I be in this research?

We expect that your taking part in this research will last 24 months.

# Why is this research being done?

The purpose of this study is to compare the safety and efficacy of the investigational VIA Disc Nucleus Pulposus (NP) allograft in participants with symptomatic degenerative disc disease (DDD) to a sham (placebo) needle injection.

# What happens to me if I agree to take part in this research?

If you agree to participate in this study, the first participant enrolled at each site (up to 20 total sites) will get the VIA Disc NP injection so we can make sure each site is ready (“Roll-in Stage”). After that, the Randomization Stage will begin, during which participants will be randomly assigned to one of the two groups. You have about a 67% chance of getting the VIA Disc NP injection and a 33% chance of getting a sham needle injection. You won’t know which one you get. You will fill out electronic surveys about your back pain, how it affects your daily life, your overall health, and your satisfaction with treatment at screening, 1, 3, 6, 12, and 24 months. You will undergo imaging of your spine (MRI, X-ray, and maybe MRS) at screening and at six months. You will follow the same visit schedule and tests as everyone else so we can compare safety and feasibility information for up to 12 months. Before your treatment procedure, please stop taking pain medicines (except acetaminophen or NSAIDs for other conditions) 48 hours beforehand.

Both the sham needle and VIA Disc NP treatment group participants will find out about their study group at the 12-month visit. At your 12-month visit, you’ll complete all surveys and find out which treatment you got. If you were in the sham group, your study doctor will let you know if you qualify to switch (“crossover”) to VIA Disc NP treatment group. If you wish to crossover, your study doctor will schedule your VIA Disc NP procedure, which will occur that same day, if you agree.

If you were in VIA Disc NP treatment group, you will be required to complete 9 follow-up visits, including in-person visits at 1, 3, 6, 12, and 24 months, and by phone at 9, 15, 18, and 21 months.

If you were in sham needle group and do not crossover, you will complete your study exit visit at 12 month and do not require further follow-up visit.

If you were in sham needle group and crossover to VIA Disc NP treatment group at your 12-month visit, you will be required to complete a total of 10 follow-up visits. This includes in-person visits at 1, 3, 6, 12 months after your sham needle injection, as well as a phone visit at 9 months. After crossing over, you will then repeat the same follow-up schedule again: in-person visits at 1, 3, 6, and 12 months, and a phone visit at 9 months after your VIA Disc NP injection.

# Could being in this research hurt me?

Transmission of disease of unknown cause and transmission of infectious agents including but not limited to HIV, hepatitis, syphilis, or microbial contaminants is possible. Continued pain may occur. Bruising, bleeding, muscular or neurologic deteriorations or bladder or bowel dysfunction may occur.

**Will being in this research benefit me?**

You may or may not benefit from participating in this research study. Your spine pain, spine function, and/or degenerative progression of your spine may or may not improve while you are in this study.

By tracking the treatments and outcomes of patients who have been treated for low back pain, health care providers can better understand how these treatments benefit their patients and improve experiences for their patients, and you may benefit if you need other treatments for your low back pain in the future.

**What other choices do I have besides taking part in this research?**

You do not need to be in this study to receive treatment for your condition. If you decide not to enter this study, there are other choices available for your treatment. These may include:

- Conservative (non-surgical) treatment (for example, physical therapy or exercise program)
- Oral pain medication
- Surgery

**What else should I know about this research?**

Other information that may be important for you to consider so you can decide whether to take part in this research is you may not be able to take some or all of the medications you have been taking for your condition or for other reasons. There is a risk of stimulation of immune responses that might keep you from receiving solid organ or hematologic transplants in the future.

**DETAILED RESEARCH CONSENT**

You are being invited to take part in a research study. A person who takes part in a research study is called a research participant.

**Why is this research being done?**

The purpose of this research study is to compare the safety and efficacy of VIA Disc NP procedure to participants with symptomatic degenerative disc disease (DDD) to participants who received a sham (placebo) procedure. Participants who receive the sham needle injection will have a procedure identical to the VIA Disc NP injection but will not be injected with VIA Disc NP and needle will only go into their muscle on the back. Participants will not know whether they receive the sham needle injection and those who do receive the sham needle injection also will have the potential to receive VIA Disc NP procedure during the study.

VIA Disc NP is a disc nucleus pulposus allograft obtained from human cadaver disc tissue. The nucleus pulposus is the soft inner core of the disc and is freeze-dried and ground to small particles. This tissue is then packaged and sterilized. VIA Disc NP is rehydrated with 2ml of sterile saline for injection. A single dose (100mg) of VIA Disc NP is injected at the affected level(s). The treatment may potentially decrease pain, improve function, and perhaps modify how fast tissue deteriorates and loses function in your back.

An allograft is the transplant of an organ or tissue from one individual to another. VIA Disc NP allograft is created from technology developed at Vivex Biologics, Inc., the study sponsor (“Vivex”). Vivex processes the allograft in a minimally manipulative way that keeps important properties for the hydration and cushioning function of the disc.

This study is designed to evaluate whether the investigational VIA Disc NP allograft is safe and effective in treating patients with low back pain by supplementing nucleus pulposus tissue in degenerated spinal discs. VIA Disc NP is processed and distributed in accordance with United States Food and Drug Administration (U.S. FDA) requirements for human cells, tissues, and cellular and tissue-based products (HCT/Ps) under 21 CFR Part 1271 regulations and the guidelines of the American Association of Tissue Banks (AATB).

This research study is being done in a group of up to 496 participants, both male and female, who are age 22 to 85 years old, like yourself with lower back pain associated with disc degeneration. These participants will be enrolled at up to 20 U.S. investigational research centers. The first participant enrolled at each site (up to 20 total) will receive VIA Disc NP procedure and the remaining up to 476 participants will be randomized with 33% chance of receiving a sham needle injection or 67% chance of receiving VIA Disc NP.

In this document, you may see the terms “treatment” and “treatment period.” These are terms used in research studies and these terms do not mean that you will be receiving medical treatment for any condition. These terms apply to the investigational study product and parts of the study where you will be receiving this study product.

**How long will I be in this research?**

Your study participation is expected to last approximately 24 months following treatment. Additional details about your participation are provided in Flowchart 1. Visit Schedule below.

**Flowchart 1**. Visit Schedule

**What happens to me if I agree to take part in this research?**

You are being asked to participate in this research study because you have discogenic low back pain unresponsive to at least 6 months of conservative care. Discogenic low back pain is low back pain that originates from a disc in your spine. If you would like to take part in the study, you will be asked to sign this consent form and complete an initial assessment visit (Screening Visit) within 90 days of study injection procedure/randomization. During these 90 days your study doctor will perform the following assessments to determine your eligibility for the study:

- Ask you about current and previous medical problems, medications and therapies.
- Conduct a physical and neurological examination and collect vital signs, which includes your height, weight, temperature, respiratory rate, pulse oximetry and blood pressure.
- Ask if you are a woman of a childbearing potential. If so, a urine pregnancy test will be performed. If you return a positive pregnancy test, you will be excluded from the study.
- Perform radiography (i.e., an X-Ray) and order a Magnetic Resonance Imaging (“MRI”) unless you have had an X-Ray and MRI of your spine within 6 months of the Screening Visit. If available to your study doctor, a Magnetic Resonance Spectroscopy (“MRS”) may be ordered.
- Ask questions to evaluate your back pain, your ability to perform daily functions, your overall health,

Once the study doctor determined that you meet all the requirements for inclusion into the study, you will be scheduled for your procedure (“Day 0”) visit. If you are one of the first 20 participants in the initial Roll-in Stage of the study, you will receive VIA Disc NP treatment. If the study is in the Randomization Stage (after first 20 enrolled), you will receive an injection of either a sham (placebo) or VIA Disc NP (investigational allograft) at up to two levels in your spine. You will be randomly assigned to one of the two groups. You have 33% chance of receiving a sham needle injection (the “sham needle group”) or 67% chance of receiving VIA Disc NP (the “VIA Disc NP treatment” group). You will not be able to choose your study group. Your study doctor who will be performing the procedure will know which group you are allocated to but your study coordinator and/or the study staff will not know your group assignment.

If you are randomized to the sham needle group, a spinal needle will be carefully inserted through the skin and muscle of the lower back at up to two levels, but no medication will be injected into your intervertebral disc by your study doctor.

If you are either one of the first 20 participants or randomized to the VIA Disc NP treatment group, you will receive an injection of VIA Disc NP (investigational allograft) at up to two levels in your spine. A needle inserted through the skin and muscle and into the center of the intervertebral disc by your study doctor.

At the procedure (Day 0) visit, your study doctor will:

- Ask you about current and previous medications and therapies.
- Conduct a physical and neurological examination and collect vital signs before and after the procedure.
- If you are a woman of a childbearing potential, perform a urine pregnancy test. If you return a positive urine pregnancy test, you will be excluded from the study.
- Complete your study treatment, either the VIA Disc NP procedure or a sham procedure.
- Assess for any Adverse Events that occurred since your last study visit.

After your treatment, you will be asked to complete follow-up assessment visits at 1, 3, 6, 12 and 24 months will include physical and neurological examinations, ask about your back pain, your ability to perform daily functions, your overall health, and your satisfaction with your back pain treatment. You will be asked about changes to your medications, therapies, and assess any adverse events that occurred since your last visit. At the six month follow-up visit only, you will be asked to complete a MRI and MRS (if available).

In between your in-person follow-up visits, you will be asked to complete 9-, 15-, 18- and 21-months assessments conducted over the telephone and/or through telemedicine collection. Telemedicine is a way to talk with your study doctor and/or member of the research staff from any place, including your home, rather than going to the research clinic or hospital. You will be asked about changes to your medications, therapies, and assess any adverse events that occurred since your last visit.

Both the sham needle and VIA Disc NP treatment group participants will find out about their study group at the 12-month visit. At your 12-month visit, you’ll complete all surveys and find out which treatment you got. If you were in the sham Needle group, your study doctor will let you know if you qualify to switch (“crossover”) to VIA Disc NP treatment group. If you wish to crossover, you will undergo the VIA Disc NP injection, which may occur that same day, if you agree.

If you were in VIA Disc NP treatment group, you will be required to complete a total of 9 follow-up visits, including in-person visits at 1, 3, 6, 12, and 24 months, and by phone at 9, 15, 18, and 21 months.

If you were in sham needle group and do not crossover, you will complete your study exit visit at 12 month and do not require further follow-up visit.

If you were in sham needle group and crossover to VIA Disc NP treatment group at your 12-month visit, you will be required to complete a total of 10 follow-up visits. This includes in-person visits at 1, 3, 6, 12 months after your sham needle injection, as well as a phone visit at 9 months. After crossing over, you will then repeat the same follow-up schedule again: in-person visits at 1, 3, 6, and 12 months, and a phone visit at 9 months after your VIA Disc NP injection.

**What are my responsibilities if I take part in this research?**

If you take part in this research, you will be responsible for completing all study assessments and study visits according to Table 1. Schedule of Assessment below.

**Table 1.** Schedule of Assessments

| Visit/Assessment | Screening | Procedure Visit | 1-Month Visit | 3-Month Visit | 6-Month Visit | 9-Month Visit (phone call) | 12-Month Visit (sham arm cross-over) | 15-Month Visit (phone call) | 18-Month Visit (phone call) | 21-Month Visit (phone call) | 24-Month Visit | Unscheduled Visit |
| --- | --- | --- | --- | --- | --- | --- | --- | --- | --- | --- | --- | --- |
|  | (0-90 days prior *) | (Day 0) | (± 7 days) | (± 14 days) | (± 28 days) | (± 14 days) | (± 45 days) | (± 14 days) | (± 14 days) | (± 14 days) | (± 60 days) |  |
| Informed Consent | X^a^ |  |  |  |  |  |  |  |  |  |  |  |
| Eligibility Criteria (Inclusion/Exclusion) | X |  |  |  |  |  |  |  |  |  |  |  |
| Demographics & Back Treatment History | X |  |  |  |  |  |  |  |  |  |  |  |
| Vital Signs ^b^ | X | X |  |  |  |  |  |  |  |  |  | X |
| Medical History & Labs (if applicable) | X |  |  |  |  |  |  |  |  |  |  |  |
| Urine Pregnancy Test (for WOCBP) | X | X |  |  |  |  | X^h^ |  |  |  |  | X^e^ |
| Physical & Neurological Exams | X | X | X | X | X |  | X |  |  |  | X | X |
| MRI & Evaluation | X^c^ |  |  |  | X |  |  |  |  |  |  |  |
| MRS & Evaluation | X |  |  |  | X |  |  |  |  |  |  |  |
| X-ray (less than or equal to 6 months from screening, AP, Lateral, Flexion-Extension) | X^c^ |  |  |  |  |  |  |  |  |  |  |  |
| VIA Disc NP Procedure |  | X^d^ |  |  |  |  | X^h^ |  |  |  |  |  |
| Oswestry Disability Index (ODI) | X |  | X | X | X |  | X |  |  |  | X |  |
| Visual Analog Scale (VAS) | X |  | X | X | X |  | X |  |  |  | X | X |
| EuroQol-5 Dimension (EQ-5D-5L) | X |  | X | X | X |  | X |  |  |  | X |  |
| Patient Global Impression of Change (PGIC) |  |  | X | X | X |  | X |  |  |  | X |  |
| Concomitant Medications | X | X | X | X | X | X | X | X | X | X | X | X |
| Opioid Use Assessment via PDMP |  |  |  |  |  |  | X |  |  |  |  |  |
| Concomitant Therapies | X | X | X | X | X | X | X | X | X | X | X | X |
| Adverse Events | X | X | X | X | X | X | X | X | X | X | X | X |
| Study Exit |  |  |  |  |  |  | X^g^ |  |  |  | X |  |

* All effort should be made to complete screening within 14 days. Up to 90 days is provided in the event of delays obtaining MRI/MRS.

^a^ Study procedures must not be performed until after informed consent is signed.

^b^ Vital Signs include oral temperature, sitting blood pressure, pulse oximetry, heart rate, and respiratory rate. On Day 0 (Day 0 Visit) Vital signs will be captured both pre & post procedure.

^c^ Participants will undergo MRI & x-rays within 6 months of screening unless site is obtaining advanced imaging, which requires new images. If greater than 6 months or not available, MRI and/or x-rays must be repeated. An MRI of the lower back per the imaging protocol must be obtained within 6 months (±28 days) of VIA Disc NP Procedure.

^d^ Participants should be observed for adverse reactions for 30 minutes post procedure prior to discharge (See Section 7.2).

^e^Urine pregnancy test should be performed if the participant is a woman of child-bearing potential and suspects pregnancy.

^f^ MRS imaging and evaluation are optional and dependent on investigational site’s capabilities and resources.

^g^ Participants who do NOT cross over at 12 months will exit the study.

^h^Sham participants with VAS ≥ 40 mm and ODI ≥ 40 may cross-over and receive the VIA Disc NP treatment at the 12-Month Visit. Any WOCBP participants who cross-over will have a urine pregnancy test performed prior to the procedure.

# Can I have other treatments during this research study?

While you are participating in this research project, your chronic pain medication including acetaminophen and non-steroidal anti-inflammatory drugs dosages will be required to remain stable or decrease throughout the trial. It is important to tell your study doctor and the study staff about any treatments or medications you may be taking, including over-the-counter medications, vitamins or herbal remedies, acupuncture or other alternative treatments. You will be allowed to continue your current treatments with the exception of those noted below. Chronic pain medications (e.g. acetaminophen) must remain stable or decrease throughout the trial. You should also tell your study doctor about any changes to these during your participation in the research project. Your study doctor will explain to you which medications need to be stopped for the time you are involved in the research project and for how long, and also what happens if your chronic pain medication needs to be increased.

During the study you will not be allowed to receive certain treatments or take certain medicines, including:

- Other injections for your back pain into your back.
- Rhizotomies (a surgical procedure to remove sensation from a painful nerve), and
- Surgery at the levels of your spine that was injected.

It may also be necessary for you to take medication during or after the research project to address side effects or symptoms that you may have. You or your insurance may be billed for these medications, so it is important that you ask your doctor about this possibility.

# Could being in this research hurt me?

****ALL SITES:** The following risk information from [START] through [END] cannot be altered without submission of supporting documentation and/or Sponsor approval of changes. Submitted changes without appropriate documentation will be reverted during Board review.

[START]

**Risks of VIA Disc NP**

The following risks and discomforts may occur in connection with the specific procedures related to VIA Disc NP. There is always the risk of very uncommon or previously unknown side effects occurring. A person may encounter some or none of the risks mentioned.

**Possible adverse events may include:**

- Bladder or bowel dysfunction
- Cerebrospinal fluid fistula
- Disorder of potency or disorder of sexual sensibility
- Epidural bleedings
- Hematoma
- Immunologic response (the possibility that a patient may develop alloantibodies should be considered for any patient who might be a future recipient of allograft tissue or cells)
- Infections (from local infections up to spondylodiscitis and/or meningitis)
- Neurological deterioration as serious as lesions of the cauda equina (paraplegia secondary to ascending bleeding or infection)
- Pain and/or inflammation
- Paresis
- Relapsing herniation
- Sensory disturbances
- Transmission of disease of unknown etiology and transmission of infectious agents including but not limited to: HIV, hepatitis, syphilis, or microbial contaminants
- Vertebral end plate inflammation

**Common (between 1 and 10 people out of 100)**

- Mild swelling or fluid within the spine following injection
- Natural progression of degenerative disc disease resumes

**Less Common (between 1 and 10 people out of 1,000)**

- Persistent swelling (swelling that continues longer than normally would be expected)

**Extremely Rare (less than 1 person out of 1,000)**

- Infections – superficial (near the level of the skin, including cellulitis, folliculitis, abscess, etc.), deep within the injected disc (septic), or systemic (bacteremia/sepsis)
- Allergic reaction or other reaction to the components of VIA Disc NP. As with any product, an allergic reaction can occur. Allergic reactions can be mild or serious and can even result in anaphylaxis and death in some cases. Common symptoms of an allergic reaction are rash, erythema, itching, swelling of the face and throat, or trouble breathing.
- Chronic back pain
- Worsening of degenerative disc disease
- Stimulation of immune responses that might keep you from receiving solid organ or hematologic transplants in the future.
- Phlebitis (inflammation of blood vessels in the leg; can cause pain and swelling)
- Pulmonary embolism (a blood clot in the lung that may cause shortness of breath or may be fatal)
- Shock (a sudden drop in blood flow throughout the body, which could lead to death)
- Reflex sympathetic dystrophy (pain, deteriorating muscle function, and skin changes)
- Nerve injury, resulting in numbness or motor weakness
- Blood vessel injury, resulting in bleeding into the skin or around the injected joint
- Disorder of potency or disorder of sexual sensibility
- Bladder or bowel dysfunction

**Risk of Getting a Disease from VIA Disc NP**

VIA Disc NP is processed from donated human cadaveric disc tissue and, therefore, a potential risk is present for the transmission of infectious disease (including but not limited to Human Immunodeficiency Virus (HIV), hepatitis, syphilis, or microbial contaminants) to the recipient participant. To minimize this risk, donors are carefully screened for diseases such as HIV and hepatitis. While the exact risk for disease transmission with VIA Disc NP is unknown, prior available data on other products reveals the risk is extremely low.

[END]

**Risks of Injection Procedure**

The following risks and discomforts may occur in connection with any injection procedure:

**Common (between 1 and 10 people out of 100)**

- Pain at the injection site
- Swelling of the back
- Warmth at the injection site
- Rash
- Itching
- Bruising and/or redness

**Extremely Rare (less than 1 person out of 1,000)**

- Infections – superficial (near the level of the skin, including cellulitis, folliculitis, abscess, etc.), deep within the injected joint (septic arthritis), or systemic (bacteremia/sepsis)
- Nerve injury, resulting in numbness or motor weakness or paresis.
- Bladder or bowel dysfunction
- Blood vessel injury, resulting in bruising or bleeding into the skin or around the injected joint
- Phlebitis (inflammation of blood vessels in the leg; can cause pain and swelling)

It is important to note that there may also be additional side effects that are not known at this time.

**Risks associated with local anesthetic:**

Local anesthesia involves numbing an area of the body using a type of medicine called a local anesthetic. Using local anesthetics doesn't usually cause problems; however, like with anything, there can be some risks. Some examples of risks are:

- Allergic Reactions: Some people might be allergic to the ingredients in the anesthetic, which could cause itching, swelling, or trouble breathing.
- Nerve Damage: In rare cases, the area where the anesthetic is injected could get damaged, causing numbness or tingling that lasts longer than expected.
- Temporary Effects: Sometimes, the anesthetic can make you feel dizzy, sick to your stomach, or give you a headache, but these usually go away pretty quickly.
- Interactions with Medications: If you're taking other medicines, the anesthetic might not work as well or could interact with them in a way that's not good for you.

It's important to talk to your doctor about any concerns you have before getting a local anesthetic, especially if you've had problems with it in the past or if you have other health conditions.

**Pregnancy-Related Risks**

This research study may involve risks to you or your unborn child, which are currently not known. Women of childbearing potential must use a type of birth control. Before entering the study, you and your study doctor must agree on the method of birth control you will use during the entire study.

Methods of birth control for this study include:

Abstinence (not having sex), if this is your usual and preferred lifestyle choice.

Hormonal (examples are birth control pills or patches, progestin implant, or injection).

Barrier method (examples are diaphragm with spermicide, intrauterine device [IUD], condom, and foam).

Women who are pregnant or nursing a child may not take part in this study. If you think that you may be pregnant during the study, you must tell your study doctor immediately.

**Risks Associated with Magnetic Resonance Spectroscopy & Imaging (MRS/MRI) Scanning**

An MRI scan will be performed at Screening (if not done within the prior 6 months or for advanced MRI technique) and at 6 months post-procedure. If greater than 6 months or not available, MRI and/or x-rays must be repeated. An MRI of the lower back must be obtained within 6 months (+ 28 days) of the injection.

MRI stands for magnetic resonance imaging. MRS stands for magnetic resonance spectroscopy. An MRI scanner is a machine that uses electromagnetic radiation (radio waves) in a strong magnetic field to create images of the inside of your spine and is also capable of MRS. Electromagnetic radiation is not the same as ionizing radiation in x-rays or computerized tomography (CT scans). The potential risks of MRI/MRS are minimal.

MRI/MRS are generally well-tolerated procedures, and your study doctor will determine whether these will cause any harm to you before any imaging is started. You will also be asked to leave all metal objects, such as keys and jewelry, in a locker outside of the scanning room. You may feel claustrophobic (fear of enclosed places) inside of a scanner because the space is small. This is a loud scanner, which is why you will be given earplugs and/or earmuffs to decrease the noise. You may feel slight tingling sensations, usually along your arms or your lower back. If you move your head while the machine is doing the scan, you may feel dizzy or nauseous.

**Risks Associated with X-rays**

X-ray evaluations will be done at Screening (if not done within the prior 6 months).

There are risks from ionizing radiation resulting from the x-ray evaluation. During the conventional x-ray examination of your lumbar spine, the effective radiation dose is approximately equivalent to 6.4 years of the natural environmental background radiation for the average U.S. citizen.

There is also some exposure to radiation associated with the image amplifying at the time of study injection. The risk from the radiation dose received from this procedure is too small to be detected. If you are especially concerned about radiation exposure, you may discuss this thoroughly with the study doctor. The lowest dose of radiation and appropriate lead shielding is used.

**Risks during study participation will be minimized by the following:**

- The investigator will receive specific training and instruction in the use of the investigational product.
- Study participants will undergo intensive monitoring by a multidisciplinary team throughout the study.
- Pre-clinical testing has been performed to optimize product safety and function.

# Will it cost me money to take part in this research?

You will not be required to pay for the injection and Vivex will provide the allograft at no cost to you. Tests and procedures that are done only for the study will not be billed to you or your insurance company.

# Will being in this research benefit me?

You may or may not benefit from participating in this research study. Your spine pain, spine function, and/or degenerative progression of your spine may or may not improve while you are in this study. The results of this study may help people with degenerative disc disease of the spine in the future.

By tracking the treatments and outcomes of patients who have been treated for low back pain, health care providers can better understand how these treatments benefit their patients and improve experiences for their patients, and you may benefit if you need other treatments for your low back pain in the future.

# What other choices do I have besides taking part in this research?

You do not need to be in this study to receive treatment for your condition. If you decide not to enter this study, there are other choices available for your treatment. These may include:

- Conservative (non-surgical) treatment (for example, physical therapy or exercise program)
- Oral pain medication
- Surgery

Ask the study doctor to discuss these alternatives with you.

# What happens to the information collected for this research?

Your private information and your medical record will be shared with individuals and organizations that conduct or watch over this research, including:

- The research sponsor
- People who work with the research sponsor
- Government agencies, such as the Food and Drug Administration
- WCG IRB, the Institutional Review Board (IRB) that reviewed this research
- The Department of Health and Human Service (DHHS)
- Other doctors, health care professionals or research staff who are involved in the study

We may publish the results of this research, however, we will keep your name and other identifying information confidential.

We protect your information from disclosure to others to the extent required by law. We cannot promise complete confidentiality.

A description of this clinical trial will be available on http://www.ClinicalTrials.gov, as required by U.S. Law. This Web site will not include information that can identify you. At most, the Web site will include a summary of the results. You can search this Website at any time.

# Who can answer my questions about this research?

If you have questions, concerns, or complaints, or think this research has hurt you or made you sick, talk to the research team at the phone number(s) listed in this document.

This research is being overseen by WCG IRB. An IRB is a group of people who perform independent review of research studies. You may contact them at 855-818-2289 or clientcare@wcgclinical.com if:

- You have questions, concerns, or complaints that are not being answered by the research team.
- You are not getting answers from the research team.
- You cannot reach the research team.
- You want to talk to someone else about the research.
- You have questions about your rights as a research participant.

# What if I am injured because of taking part in this research?

If you are injured or get sick because of being in this research, call the study doctor immediately. The study doctor will provide medical treatment. Your insurance may be billed for this treatment. The sponsor will pay any charges that are not covered by insurance policy or the government, provided the injury was not due to your underlying illness or condition and was not caused by you or some other third party. No other payment is routinely available from the study doctor or sponsor.

# Can I be removed from this research without my approval?

The person in charge of this research can remove you from this research without your approval. Possible reasons for removal include:

- It is in your best interest
- You have a side effect that requires stopping the research
- You need a treatment not allowed in this research
- You become pregnant
- The research is canceled by the FDA or the sponsor
- You are unable to take the research medication
- You are unable to keep your scheduled appointments

We will tell you about any new information that may affect your health, welfare, or choice to stay in this research.

# What happens if I agree to be in this research, but I change my mind later?

If you withdraw your permission after you have entered the study, you cannot continue participating in the study. If you refuse to give permission or withdraw your permission, your medical care and your relationship with the health care providers at the study center will not be affected.

# Will I be paid for taking part in this research?

For taking part in this research, you may be paid up to a total of up to $1,520.00. Your compensation will be broken down as follows:

- At the end of each quarter (every 3 months) you will receive by email a Visa gift card for visits you completed during that time period.
- You will receive the following amounts upon completion of each study visit at the end of each quarter:
  - Screening ($150.00)
  - Procedure (Day 0) ($150.00)
  - 1-month ($150.00)
  - 3 months ($150.00)
  - 6 months ($150.00)
  - 9 months (phone call) ($10.00)
  - 12 months ($150.00)
  - 15 months (phone call) ($10.00)
  - 18 months (phone call) ($10.00)
  - 21 months (phone call) ($10.00)
  - 24 months ($400.00)
  - Unscheduled ($150.00)

The results from this study may lead to new commercial products or tests.  If this happens you will not receive any additional compensation.

# Statement of Consent:

Your signature documents your consent to take part in this research.

Printed name of participant

Signature of participant	Date

I confirm I have explained the study to the participant and the participant has agreed to be in the study

Signature of person obtaining consent	Date

**AUTHORIZATION TO USE AND DISCLOSE INFORMATION FOR RESEARCH PURPOSES**

**What information may be used and given to others?**

The study doctor will get your personal and medical information. For example:

- Past and present medical records
- Research records
- Records about phone calls made as part of this research
- Records about your study visits

**Who may use and give out information about you?**

The study doctor and the study staff. They may also share the research information with an agent for the study doctor, if applicable.

**Who might get this information?**

The sponsor of this research. “Sponsor” means any persons or companies that are:

- working for or with the sponsor, or
- owned by the sponsor.

**Your information may be given to:**

- The U.S. Food and Drug Administration (FDA),
- Department of Health and Human Services (DHHS) agencies,
- Governmental agencies in other countries,
- The institution where the research is being done,
- Governmental agencies to whom certain diseases (reportable diseases) must be reported, and
- Institutional Review Board (IRB).

**Why will this information be used and/or given to others?**

- to do the research,
- to study the results, and
- to make sure that the research was done right.

If the results of this study are made public, information that identifies you will not be used.

**What if I decide not to give permission to use and give out my health information?**

Then you will not be able to be in this research study.

**May I review or copy my information?**

Yes, but only after the research is over.

**May I withdraw or revoke (cancel) my permission?**

This permission will be good until December 31, 2070*.*

You may withdraw or take away your permission to use and disclose your health information at any time. You do this by sending written notice to the study doctor. If you withdraw your permission, you will not be able to stay in this study.

When you withdraw your permission, no new health information identifying you will be gathered after that date. Information that has already been gathered may still be used and given to others.

**Is my health information protected after it has been given to others?**

There is a risk that your information will be given to others without your permission.

**Authorization:**

I have been given the information about the use and disclosure of my health information for this research study. My questions have been answered.

I authorize the use and disclosure of my health information to the parties listed in the authorization section of this consent for the purposes described above.

**AUTHORIZATION SIGNATURE:**

**Signature of participant	Date**
